# Affective lability as a prospective predictor of subsequent bipolar disorder diagnosis: a systematic review

**DOI:** 10.1186/s40345-021-00237-1

**Published:** 2021-11-01

**Authors:** Rosie H. Taylor, Andrea Ulrichsen, Allan H. Young, Rebecca Strawbridge

**Affiliations:** 1 Department of Psychological Medicine, Institute of Psychiatry, Psychology and Neuroscience, King’s College London, London, SE5 8AF UK; 2 South London & Maudsley NHS Foundation Trust, Maudsley Hospital, London, UK

**Keywords:** Bipolar disorder, Systematic review, Affective lability, Mood instability, Prospective, Predictor, Risk-factor, Bipolar spectrum

## Abstract

**Objectives:**

The early pathogenesis and precursors of Bipolar Disorder (BD) are poorly understood. There is some cross-sectional and retrospective evidence of affective lability as a predictor of BD, but this is subject to recall biases. The present review synthesises the prospective evidence examining affective lability and the subsequent development of BD at follow-up.

**Methods:**

The authors performed a systematic search of PubMed, PsycInfo and Embase (1960–June 2020) and conducted hand searches to identify studies assessing affective lability (according to a conceptually-inclusive definition) at baseline assessment in individuals without a BD diagnosis, and a longitudinal follow-up assessment of bipolar (spectrum) disorders. Results are reported according to the PRISMA guidelines, and the synthesis without meta-analysis (SWiM) reporting guidelines were used to strengthen the narrative synthesis. The Newcastle–Ottawa Scale was used to assess risk of bias (ROB).

**Results:**

11 articles describing 10 studies were included. Being identified as having affective lability at baseline was associated with an increased rate of bipolar diagnoses at follow-up; this association was statistically significant in six of eight studies assessing BD type I/II at follow-up and in all four studies assessing for bipolar spectrum disorder (BSD) criteria. Most studies received a ‘fair’ or ‘poor’ ROB grade.

**Conclusions:**

Despite a paucity of studies, an overall association between prospectively-identified affective lability and a later diagnosis of BD or BSD is apparent with relative consistency between studies. This association and further longitudinal studies could inform future clinical screening of those who may be at risk of BD, with the potential to improve diagnostic accuracy and facilitate early intervention.

**Supplementary Information:**

The online version contains supplementary material available at 10.1186/s40345-021-00237-1.

## Background

The chronic nature and disabling impacts of bipolar disorders (BD) are well recorded and addressed in translational research, (American Psychiatric Association 2013; Merikangas et al. 2007) but diagnosis remains delayed (for many individuals, by a decade after symptom onset) and these delays precede poorer outcomes and additional illness burdens (Lloyd et al. 2011). The distinct gaps in understanding how to predict and/or prevent BD (Woo et al. 2015) mean that there is little to offer people prior to receipt of a diagnosis. These challenges could be attenuated with the use of predictive clinical features describing bipolar signatures (Woo et al. 2015).

Newly emerging prodromal features of BD include dysregulated sleep, mood, and energy, including irritability (Correll et al. 2007; Skjelstad et al. 2010) (Trait) dysregulation of affect as a whole is also putatively associated with subsequent diagnosis of BD (Correll et al. 2007; Lish et al. 1994). Diverse terminology is used to describe various measures broadly assessing dysregulated affect, with examples including ‘mood lability’, ‘cyclothymic temperament’, ‘affective instability’ and ‘mood swings’ (Correll et al. 2007; Faedda et al. 1995; Miklowitz and Chang 2008; Rucklidge 2008). In this review, we use the term ‘affective lability’ to inclusively refer to these variable measures of extreme and alternating moods. The term affective lability is purposefully broad and is used by the present paper to encompass fluctuations of mood and emotional state, in addition to arousal/activation. A commonality between these aforementioned measures of fluctuating affect is its consideration as a *trait* construct. The nature of BD as an illness where individuals, by definition, experience switches in affect renders it plausible that (trait) fluctuating affect could be a durable preceding characteristic of people who subsequently develop BD. Affective lability will be conceptualised broadly in the present review as a ‘predictor’ or ‘precursor’ of BD without determination of a strict developmental timeline.

The relationship between affective lability and BDs which fall just outside of DSM type I/II, conceptualised as not otherwise specified (NOS), also referred to as bipolar spectrum disorders (BSD), is also worthy of review. As well as being increasingly recognised in diagnostic manuals, clinical assessment tools are also validated accordingly for BSDs (e.g. SADS) (Akiskal 1996; Angst 2013). There is further evidence of BSDs being common illnesses to BD-I and BD-II (Angst 2013) and of BSDs being used to predict diagnostic conversion to BD (Woo et al. 2015). Therefore, the present review will not limit definitions of bipolarity or BSDs.

There is also some evidence that affective lability may influence and predict the clinical course, features, and outcomes of BD or BSD after diagnosis. For example, cyclothymic temperament in BD patients has a significant impact on longitudinal functional outcomes such as impairments to home-management, social life, and leisure activities (Nilsson et al. 2012). The present review intends to consider and clarify these emerging associations.

The present review is novel in its synthesis of the existing literature incorporating an inclusive definition of affective lability and consequent inclusivity of assessment tools. Previous research and reviews assessing the relationship between affective lability and BDs have used retrospective and cross-sectional study designs (Correll et al. 2007; Egeland et al. 2000; Özgürdal et al. 2009; Leopold et al. 2012). Because these are susceptible to recall bias, the present review will only review prospective studies which used longitudinal study designs (Howes et al. 2011).

### Objectives

The primary aim of this systematic review is to establish whether people without BD, prospectively identified as having affective lability, are more likely to meet criteria for BSD/BD at a follow-up timepoint than those without affective lability at baseline. To our knowledge, this has not yet been subject to systematic review. As a secondary objective, the present review will also include and synthesise any further measures of diagnostic subtypes in BD patients whose affective lability was prospectively identified.

## Materials and methods

### Protocol and registration

The present review adheres to the preferred reporting items for systematic reviews and meta-analyses (PRISMA) statement (Moher et al. 2009). A protocol was pre-registered to the international prospective register of systematic reviews (PROSPERO 2020, registration CRD42020183945).

Initially the review registration specified that BD diagnoses should follow DSM or ICD-10 conceptualisation. Shortly after the protocol publication, and before the search had been run, it was decided that the broader BSDs have sufficient evidence of potential use in future clinical practice to be considered and reported. Results for DSM BD and BSD have been differentiated in this review. No other changes were made to the methodology after protocol registration.

### Eligibility criteria

Studies were eligible for inclusion if (i) the study design was longitudinal; (ii) human participants of any age were assessed; (iii) affective lability was measured prospectively (i.e. baseline measurement in participants not currently meeting criteria for bipolar (spectrum) disorder); (iv) a measure of affective lability, as deemed to be assessing fluctuations in mood or affect, was assessed at baseline; (vi) BD or BSD was assessed at a follow-up timepoint. Reasons for exclusion included participants having a diagnosis of borderline personality disorder (BPD) or BD at intake.

### Search strategy

Key search terms were entered into the following electronic databases: PubMed, PsycInfo and EMBASE (all dates from inception to June 2020). The search comprised the following terms: (bipolar* or psychiatric or affective disorder* or mood disorder* or psychopathology) and (mood instability or mood shift* or moodiness or cyclothymic temperament or mood lability or mood swing* or TEMPS or temperament*) and (prospective or longitud* or follow up). All generated studies were limited to those with a title and abstract available in the English language.

Two reviewers (RHT and AU) independently screened the titles and abstracts of all retrieved articles using Rayyan open-source review management software (Ouzzani et al. 2016). Reviewers were not blinded to the objectives of the review. Each article was formally screened against eligibility criteria by these two reviewers. The reviewers discussed all conflicts in study selections and a consensus was reached with the support of a third reviewer (RS). Reviewers had access to the same articles but were blinded to one another’s selections during the screening process. This process was repeated for the full-text screening of all articles selected as being potentially eligible. Reference lists of eligible papers were manually handsearched to identify further articles for screening.

### Data extraction

Reviewer RHT extracted relevant study details such as citation details, recruitment methods, sample size, follow-up duration, proportional rates of BD diagnoses, and affective lability assessment tools. Information regarding participants’ characteristics, measures, and study design were also extracted for both baseline and follow-up. Any available measure of association used to assess the relationship between affective lability and BD conversion was examined. The accuracy of data extraction was checked by a second reviewer (AU). Any disagreements were discussed in conjunction with a third reviewer (RS).

### Risk of bias assessments

The quality of all selected articles was assessed independently by RHT and AU using the Newcastle Ottawa scale (NOS) grading system for longitudinal studies in systematic reviews using a ‘star grade system’ before consensus was reached by examination of a third reviewer (RS) (Wells et al. 2012). Two reviewers (RHT, RS) were involved in establishing the assessment criterion specifications from the NOS scale, specific to this review topic, a priori. Any individual article could be awarded between zero and nine stars. As recommended in quality improvement reviews, seven stars or more is deemed a ‘good’ study, five or six stars is deemed ‘fair’, and less than five stars is deemed ‘poor’ (for more detail regarding criteria for ROB ratings, see Additional file 1) (McPheeters et al. 2012). ROB assessments were used to guide the narrative synthesis in the present review, with more emphasis given to studies with higher star-graded quality ratings.

### Analysis

Due to the heterogeneity of populations studied, measures and study designs employed, a quantitative meta-analysis was not considered appropriate. Findings and methodology across the selected articles are presented and analysed using tables and a narrative synthesis. To strengthen the narrative synthesis of results, the present review has used the synthesis without meta-analysis (SWiM) reporting guidelines for systematic reviews (Campbell et al. 2020).

## Results

### Study selection

**Fig. 1:**
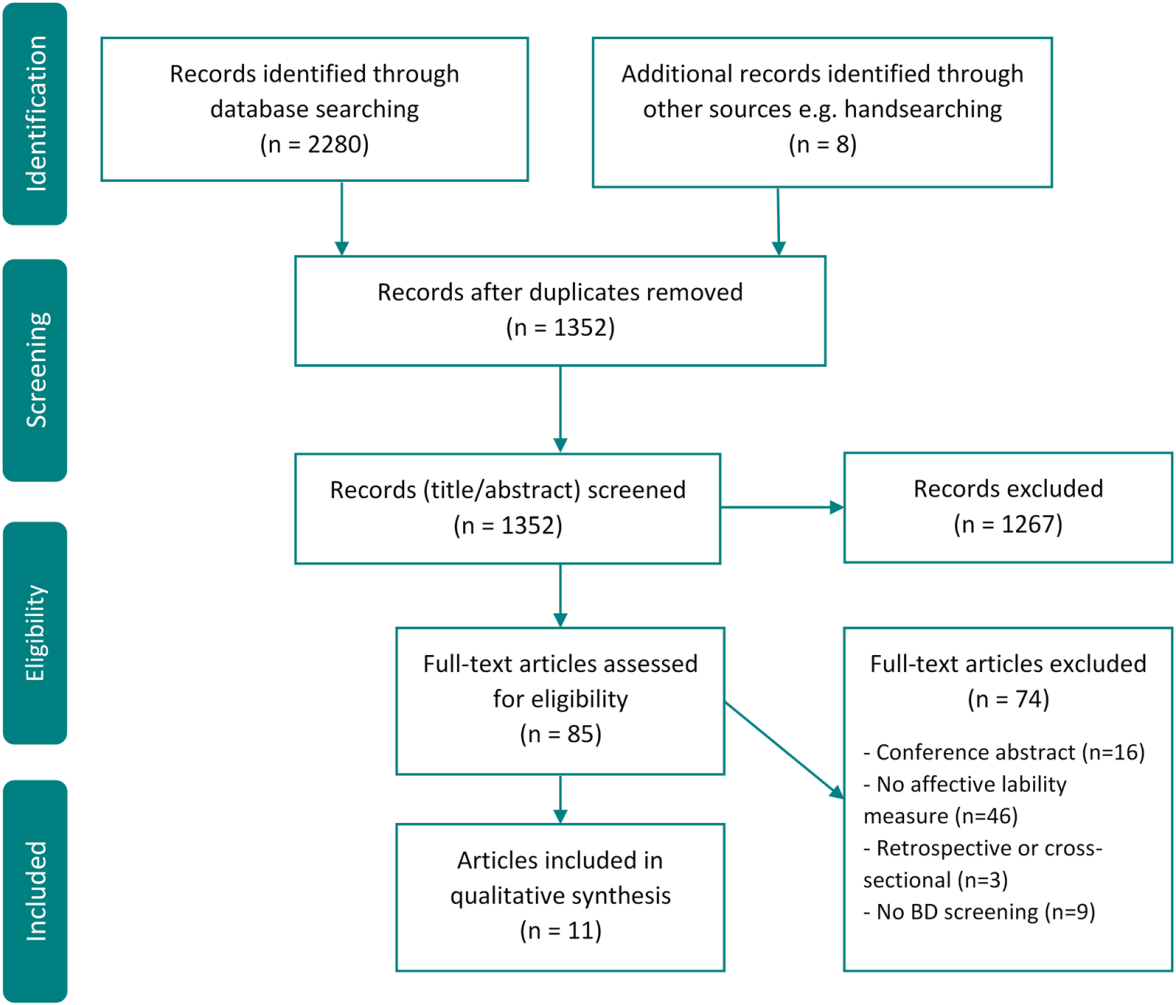
PRISMA flow diagram of the study selection process. Study flow diagram showing the Preferred Reporting Items for Systematic Reviews and Meta-Analyses. BD = bipolar disorder

As demonstrated in the PRISMA flowchart (Fig. 1), the systematic search generated 2280 records. Deduplication removed 936 and 8 further articles were identified through handsearches. 1267 studies were excluded on the basis of their abstract and titles not fulfilling eligibility criteria e.g. due to clearly not having a prospective study design, assessment of bipolarity or affective lability. The resulting 85 full texts were reviewed, with 74 being excluded, most commonly for not having an assessment of affective lability. During the screening process, authors of all conference abstracts were contacted to glean potentially relevant grey literature. No eligible studies were identified through this process. 11 articles were deemed eligible for inclusion and synthesis. The reference lists of these selected manuscripts were hand checked to identify any remaining studies by reviewer RHT; none were identified. One study was reported in two articles (DeGeorge et al. 2014; Sperry et al. 2020). These employed distinct measures, both of which fell under the present review’s inclusive conceptualisation of affective lability.

### Study characteristics

The baseline study characteristics for all included studies are presented in Table 1. All studies employed an assessment for BD at intake.

**Table 1:** Study and participant characteristics at baseline

Article	Location	Clinical Status	Age	N	Sex	Recruitment Method	Affective Lability measure	Affective Lability assessment tool	Ascertainment of affective lability
Akiskal et al. 1995	US	MDD	17 +	NR	NR	Sub-population from larger depression study	Mood Lability	GZTS (emotional stability) + MMPI (neuroticism) combined	Self-report
Angst et al. 2003	Switzer-land	Non-clinical	Range = 19–20 Mean = 20	591	F = 51%	Community cohort study	Emotional & vegetative lability	SCl-90-R	Self-report
DeGeorge et al. 2014*	US	At-risk, clinical, non-clinical	Mean = 20 SD = 2	123	F = 69%	Introductory Psychology course	Cyclothymic/irritable temperament	TEMPS-A	Self-report
Sperry et al. 2020*				101			Emotion dynamics (positive & negative affect)	ESM for PA and NA	
Egeland et al. 2012	US	At-risk offspring & non-clinical	Most < 14	221	F = 51%	Sub-population from larger study (CARE)	Mood Lability	Interview developed by expert panel	Clinician
Gan et al. 2011	China	MDD	NR	344	F = 62%	Hospital archive records, outpatient clinical records	Diurnal variation in mood	Clinical assessments	Self-report
Hafeman et al., 2017	US	Non-clinical BD offspring	Range = 6–18 Mean = 12 SD = 4	480	NR	Advertisement, research studies, outpatient clinics	Affective Lability	CALS	Self-report
Kochman et al. 2005	France	MDD	Mean = 13 SD = 3	109	NR	Child inpatient admissions	Cyclothymic-hypersensitive temperament	Adapted TEMPS-A cyclothymic scale	Self-report
Ratheesh et al. 2015	Australia	MDD, anxiety, SUD, ADD	Range = 15–25 Mean = 20 SD = 3	70	F = 85%	At-risk youth seeking mental health support	Cyclothymic temperament	TEMPS-A	Self-report
Salvatore et al. 2013	Italy, US	MDDP	Range = 10–82 Mean = 35 SD = 16	500	F = 45%	Psychiatric inpatients	Affective or psychomotor instability	AMDP and BSABS	Trained evaluator assessments
Tohen et al. 2012	US	MDDP	Range = 18–72 Mean = 36 SD = 15	56	F = 50%	Inpatient unit	Mood lability	AMDP + BABS	Research assistant

*US* United states, *MDD* major depressive disorder, *NR* not reported, *GZTS* Guilford-Zimmerman Temperament Survey, *MMPI-N* The Minnesota Multiphasic Personality Inventory, for Neuroticism, *F* female, *M* male, *SCl-90-R* Symptom Checklist-90-R, *SD* standard deviation, *TEMPS-A* Temperament Evaluation of Memphis, Pisa, Paris and San Diego Auto-questionnaire, *ESM* experience sampling method, *PA* positive affect, *NA* negative affect, *CARE *prospective Children and Adolescent Research Evaluation study, *SCID* Structured clinical interview for the Diagnostic and Statistical Manual of Mental Disorders, *BD* bipolar disorder, *CALS* Children’s Affective Lability Scale, *SUD* substance use disorder, *ADD* attention deficit disorder, *MDDP *MDD with psychosis, *AMDP *Manual for the Assessment and Documentation of Psychopathology, *BSABS/BABS *Bonn Scale for the Assessment of Basic Symptoms

^*^Sperry et al. 2020 and DeGeorge et al. 2014 are two papers from the same study. Although analyses were conducted for participants without BD at baseline, participant sex and age averages were not provided for the subgroup considered in this review (we report averages from the total sample, which are expected to be comparable)

From the 10 included studies, a range of cohorts were assessed. 5 studies recruited non-clinical cohorts (DeGeorge et al. 2014; Sperry et al. 2020; Hafeman et al. 2017; Angst et al. 2003; Egeland et al. 2012). However, each of these non-clinical cohorts had been screened for a factor that put them at risk, such as having a family history of BD. The other 5 studies assessed clinical cohorts, 4 with participants who had MDD (with or without psychotic features) and 1 recruiting a mix of individuals with MDD, anxiety or substance use disorders (Ratheesh et al. 2015). Recruitment methods varied and included using inpatients (Tohen et al. 2012; Salvatore et al. 2013; Kochman et al. 2005), outpatients, hospital records and advertising (Hafeman et al. 2017; Angst et al. 2003; Ratheesh et al. 2015; Gan et al. 2011), sub-populations from larger studies (Egeland et al. 2012; Akiskal et al. 1995) and psychology students (DeGeorge et al. 2014; Sperry et al. 2020).

A wide range of tools were employed to assess affective lability. Three studies (DeGeorge et al. 2014; Ratheesh et al. 2015; Kochman et al. 2005) used validated versions of the Temperament Evaluation of Memphis, Pisa, Paris and San Diego Auto-questionnaire (TEMPS-A) (Akiskal and Akiskal 2005; Vázquez and Akiskal 2005). Terminology used to describe unstable and alternating moods varied. The term ‘mood lability’ was used by three studies, employing different assessment tools (see Table 1). Other terminology used included ‘diurnal variation in mood’ (n = 1), ‘cyclothymic temperament’ (n = 1), ‘emotional and vegetative lability’ (n = 1) ‘affective lability’ (n = 1), ‘affective or psychomotor instability’ (n = 1), ‘cyclothymic/irritable temperament’ (n = 1), ‘cyclothymic-hypersensitive temperament’ (n = 1), and ‘emotional instability’ (n = 1).

Study characteristics at follow-up are reported in Table 2. Most studies followed up a participant pool of more than 100 participants (n = 7). Follow-up durations ranged from 1 year (Ratheesh et al. 2015; Gan et al. 2011) to 16 years (Egeland et al. 2012). Diagnoses of BD or BSD were provided by clinicians (n = 6) or trained interviewers (n = 4) and the number of conversions to BSD/BD ranged from four (Sperry et al. 2020; Ratheesh et al. 2015) to 86 (Angst et al. 2003) individuals. The proportion of the sample who converted ranged from 4% (Sperry et al. 2020; Egeland et al. 2012) to 43% (Kochman et al. 2005). Diagnostic tools also varied, as presented in Table 2.

**Table 2:** Study characteristics and findings at follow-up

Article	FU n	% lost at FU	FU duration (years)	Diagnostic Tool	Diagnostic assessor	Diagnosed with BD n (%)	Rates of BD type	Affective lability/BD association
Akiskal et al. 1995	559	NR	11	SADS (DSM /RDC)	Clinician	70 (13)	22 BD-I, 48 BD-II	BD-II X^2^ = 19.92 + + + (specificity = 86%, sensitivity = 42%)
Angst et al. 2003	591	NR	15	DSM-IV criteria	Clinician	86 (15)	41 BD-II, 45 BSD	BD-II + + BSD OR = 3.4, 95% CI [1.7, 6.6] + +
DeGeorge et al. 2014*	112	23%	3.1 (SD = 0.5, range 1.7—4.8)	SCID	Advanced grad (81%), psychologist + undergrad (19%)	13 (14)	BSD and BD BSD	BSD OR = 2.99 + BD OR = .532, CI [.08–3.45]
Sperry et al. 2020*	108	22%				4 (4)		PA: OR = 1.91, 95% CI [1.14, 3.18] + NA: OR = 1.54, 95% CI [1.00, 2.38] +
Egeland et al. 2012	221	NR	16	Adapted K-SADS, clinical records	Clinician	9 (4)	All BD-I	Mood lability more present in BD at risk sample than controls (p = 0.063)
Gan et al. 2011	268	22%	1	SCID-I	Psychiatrist	27 (24)	2 BD-I, 25 BD-II	OR = 0.487 +
Hafeman et al. 2017	412	14%	Mean = 8.34	K-SADS (DSM-IV)	Trained interviewers + psychiatrist review	44/299 at-risk (15)	15 BD-I/II, 29 BSD	X^2^ = 4.00 +
Kochman et al. 2005	80	27%	2–4 (27 months, SD = 9 months)	K-SADS	Investigator	35 (43)	All BSD	Prior instability in 64% of BSD; BSD vs non-BSD difference + + +
Ratheesh et al. 2015	52	26%	1	LIFE	Unspecified	4 (8)	3 BD-II, 1 BD-NOS	SES = 0.27 (p = 0.13), 95% CI [0.00,0.59]
Salvatore et al. 2013	107	79%	Mean = 4	SCID	Blinded investigator	20 (19)	10 BD-I, 10 BD-NOS	RR = 1.45 +
Tohen et al. 2012	49	13%	4	SCID	Blinded experienced raters	14 (33)	BD-I or BD-NOS	X^2^ = 4.85 +

*FU*  follow-up, *n* number of, *BD* bipolar disorder, *BPSD* bipolar spectrum disorder, *DSM* Diagnostic and Statistical Manual of Mental Disorders, *NR* not reported, *SADS* The Schedule for Affective Disorders and Schizophrenia, *RDC* the Research Diagnostic Criteria, *BPI* Bipolar Disorder Type 1, *BPII* bipolar disorder type 2, *DSM-IV* the fifth edition of the Diagnostic and Statistical Manual of Mental Disorders, *BSD* bipolar spectrum disorders, *OR* Odds ratio, *CI* Confidence intervals, *SD* standard deviation, *SCID *Structured Clinical Interviews for DSM, *PA* positive affect, *NA* negative affect, *K-SADS *Kiddie Schedule for Affective Disorders and Schizophrenia, *SCID-I* Structured Clinical Interview for the fifth edition of the Diagnostic and Statistical Manual of Mental Disorders for Axis I Disorders, *LIFE* The Longitudinal Interval Follow-up Evaluation for DSM IV, *BD-NOS* Bipolar disorder not otherwise specified, *SES* standardised effect size. *RR* Risk Ratio

^*^Sperry et al. 2020 and DeGeorge et al. 2014 are two papers from the same study. In both papers ‘% lost at FU’ and ‘FU duration’ are reported for the participant pool including some participants with initial BD diagnoses (we report averages from the total sample, which are expected to be comparable)

+ p < 0.05, +  + p < 0.01, +  +  + p < 0.001

### Primary outcome

As presented in Table 2, six studies reported a statistically significant association between prospectively-identified affective lability and a later diagnosis of BD as defined by the DSM (Hafeman et al. 2017; Angst et al. 2003; Tohen et al. 2012; Salvatore et al. 2013; Gan et al. 2011; Akiskal et al. 1995). One small study of 70 participants with a mixture of diagnoses at baseline (depression, anxiety, substance use disorder and attention deficit disorder) did not find a statistically significant association with BD (p = 0.13) although only 4 conversions to BD were recorded (Ratheesh et al. 2015). The other (Egeland et al. 2012) reported that mood lability tended to be more frequent in those at-risk for BD than controls who were not at risk but also had a low conversion rate to BD (n = 9) and as such did not undertake statistical analyses for this comparison.

Four articles reported findings for the analyses of BSD, with all identifying statistically significant relationships with this diagnosis (DeGeorge et al. 2014; Sperry et al. 2020; Angst et al. 2003; Kochman et al. 2005). Two articles reporting results for prospectively identified BSD were drawn from the same study, with each paper independently reporting distinct findings with different measures of affective lability (DeGeorge et al. 2014; Sperry et al. 2020).

Therefore, across all studies, nine of 11 articles reported a statistically significant relationship with BD/BSD.

### Secondary outcomes

As a secondary objective, we planned to explore any further BD-related clinical associations with prospectively identified affective lability. In our review protocol, the examples considered were symptom severity, episode frequency or BD diagnostic subtype pending data availability. The former two examples were not synthesisable given limited reporting and heterogeneity of included studies, however we were able to examine the type of BD diagnosis. Only one study reported on the independent statistical analyses of BD-I and BD-II, finding a significant association for the latter but not the former (Akiskal et al. 1995).

No studies only assessed BD-I. Statistically significant associations were identified where: all developed BD-I or Bipolar Disorder Not Otherwise Specified (BD-NOS), (Tohen et al. 2012) there was an even split between BD-I and BD-NOS, (Salvatore et al. 2013) a preponderance of BD participants met criteria for BD-II, (Gan et al. 2011) a preponderance met criteria for BD-NOS, (Hafeman et al. 2017) and where only BSD was considered (Sperry et al. 2020; Kochman et al. 2005). The only non-significant associations were in studies with very low conversion rates to any BD type (Egeland et al. 2012; Ratheesh et al. 2015).

### Risk of bias

Quality assessments were carried out for all 11 eligible articles and presented in Table 3. Most studies’ ROB was deemed ‘fair’ (n = 6). The highest star grading which classified as ‘good’ (7 stars) was given to only one study ﻿(Hafeman et al. 2017). A star grade of six (‘fair’), was also given to only one study (Akiskal et al. 1995). Both studies found a significant relationship between prospectively identified affective lability and later diagnoses of BD. Five studies received a star grading of five (‘fair’), of which four reported significant associations (Angst et al. 2003; Tohen et al. 2012; Salvatore et al. 2013; Gan et al. 2011) and the other reported having too low a conversion rate to undertake statistical analyses (Egeland et al. 2012). Four studies received a star grading of three and were and were therefore deemed ‘poor’, of which three identified significant associations (DeGeorge et al. 2014; Sperry et al. 2020; Kochman et al. 2005) and one did not but had a very low bipolar conversion rate (Ratheesh et al. 2015). No studies recruited cohorts which can be deemed representative of the average person without BD in the community. However, all studies screened for psychopathology at baseline (n = 11).

**Table 3:** Risk of bias assessment of included studies

ROB scale and accepted criteria	Akiskal et al. 1995	Angst et al. 2003	Egeland et al. 2012	Gan et al. 2011	Hafeman et al. 2017	Ratheesh et al. 2015	Salvatore et al. 2013	Tohen et al. 2012	DeGeorge et al. 2014	Kochman et al. 2005	Sperry et al. 2020
Selection											
Exposed cohort is representative of the average person without BD	–	–	–	–	–	–	–	–	–	–	–
Non-exposed cohort is drawn from the same community as the exposed cohort	*	*	*	*	*	*	*	*	*	*	*
Exposure ascertained through secure record or structured interview	–	–	*	*	–	–	*	*	–	–	–
Demonstration that outcome of interest was not present at start of study	*	*	*	*	*	*	*	*	*	*	*
Comparability											
Study controls for family history of BD or additional factor	**	*	–	–	**	–	*	–	–	–	–
Outcome											
Assessment of outcome uses structured clinical assessment or record linkage	*	*	*	*	*	*	*	*	*	*	*
Follow-up long enough for outcome to occur (5 + years)?	*	*	*	–	*	–	–	–	–	–	–
Adequacy of follow up of cohorts (> 80% follow up or description provided of those lost)	–	–	–	*	*	–	–	*	–	–	–
Total (max = 9)	6	5	5	5	7	3	5	5	3	3	3

Risk of bias (ROB) assessment for all included studies, using the Newcastle–Ottawa Scale for cohort studies. The criteria employed for ROB ratings are described in text and Additional file 1

^*^Method accepted. – Method not accepted

## Discussion

This systematic review set out to determine whether prospectively identified affective lability in cohorts without BD was associated with subsequent diagnoses of bipolar (spectrum) disorders. All selected and synthesised studies are prospective, making them less susceptible to recall bias. Studies are further strengthened by their use of validated diagnostic BD assessments at both baseline and follow-up.

The present systematic review revealed a reasonably consistent, positive association between prospectively identified affective lability and follow-up BD diagnoses, with 9 out of 11 studies finding a significant association with BD/BSD. For DSM-defined BD, significant associations were identified in 6/8 studies, with the remaining two having extremely small BD sample sizes, which precluded statistical analyses (Egeland et al. 2012) or led to a non-significant association (Ratheesh et al. 2015). The strength of this result is limited by the fact that most ROB assessments were ‘fair’. If anything, the relationship between prospectively identified affective lability and follow-up diagnoses of broader BSD diagnoses appears stronger, being identified in all four studies examining this (DeGeorge et al. 2014; Sperry et al. 2020; Angst et al. 2003; Kochman et al. 2005). However, 1/4 studies received a ‘fair’ ROB rating, and all others received ‘poor’, weakening the strength of this result.

Whether affective lability differentially predicts one type of bipolar illness from another is unclear. Proportional rates of various BD diagnoses were identified and have been reported in Table 2. Only one study reported on the independent statistical analyses of BD-I and BD-II, finding a significant association for the latter but not the former (Akiskal et al. 1995). This corresponds to cross-sectional findings which associate temperamental instability with BD-II disorder more so than BD-I (Akiskal et al. 2003). Despite this, there is not enough relevant data in the present review to draw firm conclusions. Furthermore, although this study received one of the highest ROB assessments in the present review, this grading was only classed as ‘fair’. This finding may also not be specific to bipolar disorders, and affective lability may commonly be a risk factor for developing other mood or personality diagnoses, something that was not in scope of the current review but clearly deserving of future evidence synthesis.

The secondary objective of this review also sought to explore any further clinical implications of prospectively identified affective lability (such as, for example, the experience of rapid cycling, mixed affective episodes, preponderance of mania or depression, or comorbid anxiety). These are all putative correlates of affective lability but no further clinical outcome measures were examinable in the current review.

There were several limitations of the present review. The first is that only 10 studies, with 11 sets of analyses, were included for review. This is likely attributable due to methodological and logistical challenges of undertaking long-term longitudinal studies. The quality and risk of bias of studies is also a limiting factor, with most included studies rated as ‘poor’ (n = 7) or ‘fair’ (n = 4). Only one study was ‘good’.

Although the reviewed measures of affective lability are deemed relatively consistent, conclusions could have been stronger if the field used a consistent, well-validated measure across all studies. ROB assessments were also graded down by low follow-up rates. Participant loss to follow-up rates is a reasonable and common limitation of long-term studies, participants who developed mental health difficulties might have been more likely to discontinue the study and represents a potential confounding factor. Low conversion rates to BD limit the strength of analyses. Diagnostic rates were likely to have been even lower had the studies not selected at-risk cohorts. However, all studies selected their samples in this way, limiting how easily the results can be generalised to the general population.

Many studies did not follow up participants for longer than two to three years. Many participants might have developed BD after this timeframe and the analyses would not represent these participants. Although these factors collectively graded most ROB assessments down, low gradings do not necessarily reflect low quality studies. Further, many studies did not assess for affective lability as a primary objective. It is therefore not surprising that this was not carefully controlled for or measured in a structured interview. The possibility of publication bias affecting these associations also cannot be disregarded.

Other methodological differences between the studies should be considered as our findings combined samples of different ages (baseline age ranging from pre-adolescence to adults) and with different types of risk factors (e.g. synthesising those with familial risk and clinical populations). However, the consistency of the associations reported in spite of these variabilities supports its potential as a valid risk factor.

The identification of stable risk factors such as affective lability, is critical to developing accurate at-risk predictive models that have the potential to consequentially improve illness detection, intervention and ultimately prognosis. Recently developed instruments for the early detection of BD include the BAR-criteria, BPSS, EPIbipolar and SIBARS (Leopold et al. 2012; Bechdolf et al. 2012; Correll et al. 2014; Fusar-Poli et al. 2018). Existing measures of bipolarity, such as the bipolarity index, also hold potential as tools for identification of those who are at-risk (Aiken et al. 2015). There is some preliminary evidence distinguishing mood lability as a precursor specific to BD when compared to schizophrenia (Correll et al. 2007). These distinctions require further clarification (Howes et al. 2011).

Clinical implications of predictive models may include minimising delays to diagnosis and improved early intervention; (Hafeman et al. 2017) BD patients respond better to medication if they receive it earlier in their illness course (Beesdo et al. 2009; Swann et al. 1999). It has even been suggested that comprehensive and reliable predictive models could also contribute to preventing the development of a full disorder (Skjelstad et al. 2010). Predictive models can help to advance this new field of research by improving the identification of appropriate non-clinical cohorts for research. For these reasons, researchers have argued that clinically applicable predictive assessment tools have the potential to transform the treatment, clinical outcomes, and illness progression of BD (Skjelstad et al. 2010; Malhi et al. 2014). However, it is important to emphasise that clinical implementation of these risk-prediction tools is challenging e.g. ethically in informing individuals that they are at-risk for BD, the potential for premature treatment initiation, identifying false positive cases, eliciting self-stigmatization. Thus, care and consideration is required not only in relation to the sensitivity and specificity of such a tool (to maximise true positive and minimise false positive cases) but also how its implementation would be managed in terms of supporting the wellbeing of individuals with being informed they are at risk, particularly relating to stigma and intervention.

There are specific cohorts who could particularly benefit from improved clinical screening bolstered by an understanding of precursor features. This will be critical for those who face BD, schizophrenia, BPD, or MDD misdiagnoses. For example, many of those with BD can initially experience years of depressive episodes without the occurrence of mania or hypomania (Bowden 2001). This can lead to a lack of treatment or being misdiagnosed with MDD. Patients can consequently receive inappropriate treatment which may exacerbate bipolar symptoms. Crucially, for those whose BD begins with depressive episodes, more severe long-term clinical outcomes, such as higher rates of suicide, have been reported (Correll et al. 2007; Baldessarini et al. 2014).

Further research into predictive features could improve the identification and treatment of BD-II. Although there is not enough research for the present review to draw conclusions regarding BD subtypes, hypomanic episodes do not always result in hospitalisation and can easily be missed, making the diagnosis of BD-II particularly challenging. This could contribute to the relatively high rates of suicide attempts and completed suicides that occur in affected patients (Rihmer and Pestality 1999). Similarly, precursor features could distinguish the pathogenesis of treatment-resistant depression from DSM-defined MDD, with potential to bring to light a broader spectrum of illness and unacknowledged features of bipolarity.

## Conclusions

The present review has found that prominently, longitudinal studies demonstrate a significant association between prospectively identified affective lability in cohorts without BD and subsequent diagnoses of BD or BSD. This pattern was observed across 9 of the 10 studies reviewed. Future studies should aim to establish this pattern using larger sample sizes, in samples which are more representative of the general population and use longer follow-up durations. Whether affective lability leads to a particular type of bipolar illness and/or trajectory should also be determined, particularly since the present review employed an inclusive approach to bipolar diagnoses. More broadly, affective lability should be further explored using well-validated measures, in combination with a range of other possible precursors in order to develop clinical predictive models and contribute to early intervention efforts, accurate diagnosis, and improved outcomes for those with BD.

## Supplementary Information

**Additional file 1**: The Newcastle Ottawa scale (NOS) grading system tailored criteria.

## Data Availability

Please make any requests to the corresponding author.
