# Supplementary material for: Affective lability as a prospective predictor of subsequent bipolar disorder diagnosis: a systematic review
Source: Int J Bipolar Disord. 2021 Nov 1;9:33. doi: 10.1186/s40345-021-00237-1 (PMC8558129; doi:10.1186/s40345-021-00237-1)
Supplement: Supplementary file 1 — Additional file 1: The Newcastle Ottawa scale (NOS) grading system tailored criteria. [file 40345_2021_237_MOESM1_ESM.docx]

**Appendix:** The Newcastle Ottawa scale (NOS) grading system tailored criteria

| **ITEM TO BE RATED ON** | **EXPLANATION OF CRITERIA USED** |
| --- | --- |
| Exposed cohort is representative of the average person without BD. | * If not a particular subgroup. No star if non-representative e.g. depressed, teenage, at-risk. |
| Non-exposed cohort is drawn from the same community as the exposed cohort. | * If no difference between recruitment of those with vs without affective lability. |
| Exposure ascertained through secure record or structured interview. | * If affective lability measurement was rated objectively, e.g. by psychiatrist or interviewer. No star if self-report or method not given. |
| Demonstration that outcome of interest was not present at start of study. | * If confirmation / assessment to show they did not meet criteria for bipolar at baseline. |
| **Comparability** |  |
| Study controls for family history of BD or additional factor. | * If comparisons between exposure (lability) and outcome (bipolar) tested for at least one confounder such as family history.  ** if more than one confounder is controlled for. |
| **Outcome** |  |
| Assessment of outcome uses structured clinical assessment or record linkage. | * A structured & validated measure of BD or BSD at outcome. |
| Follow-up long enough for outcome to occur (5+ years)? | * If 5+ years. |
| Adequacy of follow up of cohorts (>80% follow up or description provided of those lost. | * If >80% followed up (less than 20% lost to follow up). |
